# Accident due to Incorrect Selection of Diode Laser Technique in the Treatment of Oral Venous Malformation

**DOI:** 10.1155/2023/8817274

**Published:** 2023-12-08

**Authors:** Mohamed Mohsen, Guido Migliau, Daniele Pergolini, Umberto Romeo, Gaspare Palaia

**Affiliations:** Department of Oral and Maxillofacial Sciences, “Sapienza” University of Rome, Rome, Italy

## Abstract

Venous malformation (VM) originates from a congenital venous network disorder, configuring about 40% of the vascular anomalies that occur in the head and neck region. The usage of diode lasers in the treatment of VM is associated with various advantages, such as short operating time and few postoperative complications. Nevertheless, for larger and deeper VM treatment, it may require more than one session for the complete resolution of the lesion. Laser surgery for oral VM may induce heat accumulation due to excessive irradiation causing adverse events postoperatively, including pain and ulcer formation with scarring. In this clinical case, it was recommended to use the leopard technique (LT) to reduce the lesion size, evaluate the remaining unhealed lesion, and apply different laser techniques to obtain the complete resolution of the lesion.

## 1. Background

This work has been written to focus attention on the need for the correct execution of laser therapeutic protocols. In fact, the laser in dentistry can be a real resource but under proper control of the execution technique. However, our work also highlights how much any adverse effects during laser-assisted procedures can be reversible and less serious than the drawbacks during traditional dental procedures.

## 2. Case Presentation

Vascular anomalies incorporate many lesions, ranging from simple birthmarks to large, disfiguring tumors. They represent unique diagnostic and therapeutic challenges that require adequate management as they may affect swallowing, respiration, and speech.

Mulliken and Glowacki, in 1982, introduced a vascular anomalies classification according to endothelial characteristics. It was a binary classification where hemangiomas are characterized by increased proliferation and hyperplasia, while malformations demonstrate vessel dysplasia with normal rates of cell turnover [1].

The International Society for the Study of Vascular Anomalies (ISSVA) developed and revised a classification system that includes histologic features, anatomic, and embryologic, integrating genetic advances in the interpretation of vascular anomalies [2, 3].

Vascular malformations are congenital disorder that occurs due to a malformed vessel development and morphogenesis. These lesions, present at birth, grow simultaneously and never regress. They can be simple or combined lesions. Consequently, they are divided into low- and high-flow lesions [4].

It is typical in venous malformation (VM) that low-flow peripheral enhancement progresses to the center. Rapid enhancement throughout the lesion characterizes the arterial malformation, while in arteriovenous malformation, it is accompanied by flow voids [5].

VM originates from a congenital venous network disorder, configuring about 40% of the vascular anomalies that occur in the head and neck region. Common complications comprise ulceration and bleeding.

The lesion color ranges between red and purple, depending on the location, depth, and degree of vascular congestion of the affected area. It has soft consistency on palpation and can manifest as a flat or raised lesion with a smooth or nodular surface, sessile or pedunculated, with defined edges [6].

In recent years, there has been an increased consideration of sonography in the dermatologic field, where ultrasound imaging plays an important role to study lesions in the oral regions such as the tongue, oral cavity, and lips [7].

For the assessment of oral vascular lesions, many imaging modalities are available such as computed tomography (CT), magnetic resonance imaging (MRI), angiography, and ultrasonography (US). For oral and maxillofacial surgical fields, CT and MRI are the standard imaging procedures. All imaging modalities may be used to determine the extent of the lesion; however, the velocity of blood flow may be better determined by contrast-enhanced MRI and color Doppler US [8].

The color Doppler US evaluation can be used to identify feeding and draining vessels and determine the presence, quantity, and type of the Doppler flow.

Alternative treatment methods are mentioned in the literature, including surgical excision, laser surgery, cryotherapy, chemotherapy agents, corticosteroids, embolization, and sclerotherapy [9].

The introduction of laser surgery in the last two decades represented a significant innovation in several oral surgical procedures, compared to conventional scalpel surgery.

The technological advancement in laser-based treatments has minimal side effects and various benefits, such as the reduced necessity of anesthesia, precise cutting, no bleeding risk, less postoperative discomfort due to the biostimulation effect, and better healing, in addition to the needless use of sutures which are superficial with a bloodless operative field [10].

The laser tissue interaction occurs due to the tissue absorption of the energy. For an effective treatment, the laser must penetrate deeply into the targeted vessel. Additionally, the exposure needs to be long enough to cause sufficient coagulation of these vessels [11].

It is necessary to choose a laser wavelength well absorbed by hemoglobin, whereas the most effective laser wavelengths are KTP, diode laser, and Nd:YAG.

Laser surgery can be performed by various techniques, one of which is transmucosal thermocoagulation (TMT), where irradiation is done for the entire lesion and the laser fiber does not contact the tissue. In the leopard technique (LT), multiple pulsed spot irradiation is performed using a defocused beam. On the other hand, in intralesional laser photocoagulation (ILP), laser energy is delivered directly into the lesion through the fiber. ILP can be combined with LT for large lesions of thickness > 1 cm.

To perform safe laser therapy, the laser-induced heat should be controlled, and the mucosa that covers the lesion should be protected to prevent consequent side effects and overheat accumulation [12].

## 3. Case Report

An 18-year-old male was referred to the Department of Oral Surgery and Maxillo-facial Sciences of the Sapienza University of Rome with the chief complaint of an oral bluish-colored lesion on the upper lip with no history of bleeding or discharge. It was reported that the lesion appeared within the last year.

A thorough examination including the patient's details, medical and dental history, extraoral examination, and photographs was carried out. The general health condition of the patient was excellent, with no systemic manifestations.

**Figure 1:**
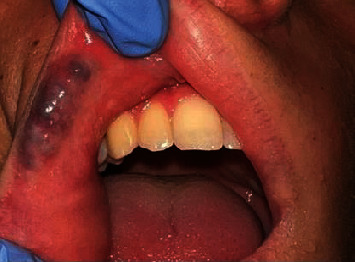
Clinical aspect of venous malformations on the upper lip.

The oral mucosa examination revealed a bluish-colored lesion (~25 × 8 mm) on the upper lip (Figure 1), elevated with well-defined margins. During palpation, the lesion was soft and whitened with applied pressure.

**Figure 2:**
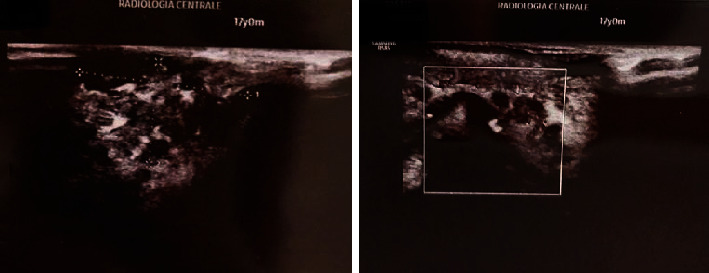
Color Doppler US imaging confirms the diagnosis of venous malformation.

A color Doppler US evaluation was prescribed and revealed that the VM was superficial, and the diagnosis of venous malformation was confirmed (Figure 2).

TMT was performed for the whole lesion at the same session, in a noncontact mode using a transparent glass slide to compress and reduce the lesion thickness. The irradiation was performed for continuously 90 seconds using these laser parameters (940 nm Laser Epic 10, Biolase, Irvine, CA, USA) in 1.5 W, comfort pulse 2 : 1 ms ON/1 ms OFF, 50% duty cycle, tip e, and diameter of 400 *μ*m.

**Figure 3:**
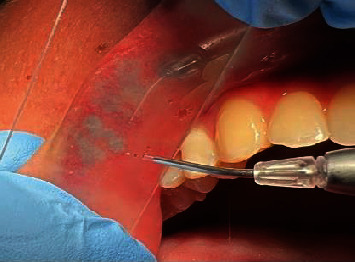
The whole lesion was treated by TMT using diode laser 940 nm.

**Figure 4:**
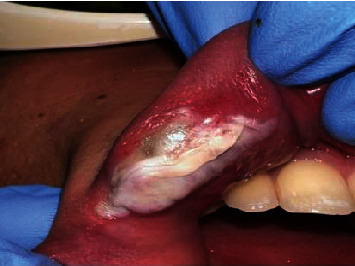
Immediately postoperative.

No cold pack was applied postoperatively, and minimal discomfort was reported (Figures 3 and 4). A written informed consent was obtained from the patient before the operation.

Only 0.2% chlorhexidine spray (Corsodyl, GlaxoSmithKline Consumer Healthcare S.p.A, Baranzate, Milan, Italy) was prescribed for 3 applications/day for 1 week.

**Figure 5:**
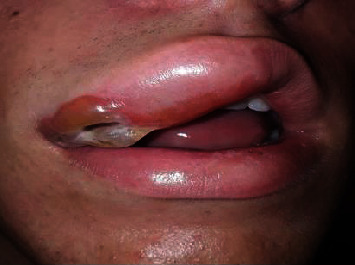
Clinical aspect after 24 hours showed swelling, edema, and ulcer formation.

The patient came back to our observation, after 24 hours, suffering from severe pain, swelling, and a deep ulcer (Figure 5).

Antibiotics (amoxicillin+clavulanic acid) at 2 g/day and analgesics were prescribed for 7 days.

Betamethasone was prescribed for 4 consecutive weeks: 4 mg/day in the first week, 3 mg/day for the second week, 2 mg/day for the third week, and 1 mg/day for the fourth week. Hyaluronic acid gel and an antiseptic spray were prescribed for 3 applications/day for 3 weeks.

**Figure 6:**
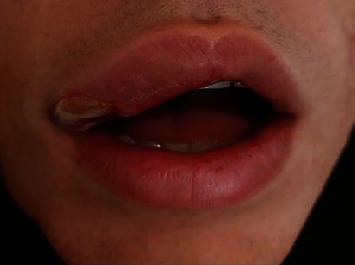
Clinical aspect after 2 weeks.

A strict follow-up was performed weekly, and an improvement was observed in each one (Figure 6).

**Figure 7:**
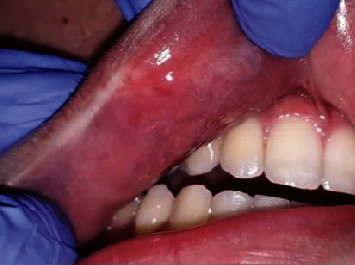
Clinical aspect after 3 months showed complete healing with minimal scar.

**Figure 8:**
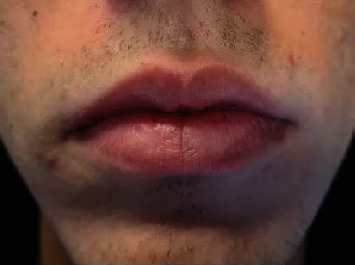
The aesthetic aspect after 3 months.

The lesion was completely healed with minimal scar formation within a month, and no recurrence was observed after 3 months (Figures 7 and 8).

## 4. Discussion

Vascular anomalies incorporate many lesions, ranging from simple birthmarks to large, disfiguring tumors. Several classifications have been introduced for vascular anomalies [13, 14]. The 2014 ISSVA guidelines can be considered the most extensive classification of vascular anomalies [2].

Laser therapy can play an important role in the treatment of vascular malformations. Frequently used lasers are diode laser (800–980 nm), KTP laser (532 nm), and Nd: YAG laser (1064 nm) [15].

There are several advantages and disadvantages of laser therapy. Advantages such as dry operating field, the minimal requirement for local anesthesia, excellent visibility, reduced postoperative pain, scaring and minimum swelling, and less mechanical trauma. Therefore, antibiotics postoperatively are reduced.

On the other hand, the disadvantages are prolonged postoperative healing due to the sealing of blood and lymphatic vessels in the surgical field and delayed wound epithelialization.

The diode laser energy turns into heat when absorbed by hemoglobin, resulting in coagulation. The penetration depth of the 980 nm wavelength appears to be lower than in the Nd:YAG laser, making it more effective in the coagulation of superficial and interstitial lesions.

Histological studies conducted on diode laser application on the vein walls revealed that coagulative necrosis causes tissue distortion not only in the vessel's inner lining epithelium but also in the outer layers of the vascular wall.

Diode laser can easily treat VM of small dimensions regardless of the wavelength. After laser therapy, VM stops growing, with a low rate of adverse reactions and no sign of recurrence.

The diode lasers have a deep penetration energy. Consequently, the laser-tissue interaction is not instantly evident. Due to its heat accumulation, laser irradiation can develop excessive destruction of the tissue. To avoid these side effects, different irradiation techniques were developed such as short-pulsed irradiation and using cooling devices.

Romeo et al. affirm that lasers are the gold standard in the treatment of VM of the oral cavity. They implied that laser parameters such as pulse duration, spot diameter, and energy density need to be evaluated for a correct laser treatment. A shorter pulse duration is preferred for small-diameter vessels while for large-diameter vessels long pulse duration must be utilized. Regarding the spot diameter, the selection should be based on both the depth and size of the lesion. As for the energy density, it should be based on the color of the lesion, since purple and bluish lesions absorb laser energy more than pink or red ones.

Oral wounds often tend to be neglected due to their fast healing with minimal scar formation compared to skin wounds.

LT is a multiple-pulsed spot irradiation technique, using a defocused laser beam that irradiates separated spots to avoid excessive heating of the tissue. It can provide a safe treatment that is less invasive than using the laser in continuous mode irradiation [16, 17].

Miyazaki et al. found that combining LT and ILP in the treatment of the massive lesions' superficial and deep layers could achieve an effective resolution of the lesion [18].

TMT is performed by irradiating the entire lesion without the laser fiber contacting the tissue. The preferred distance between the lesion surface and the laser pointer should be between 2 and 3 mm, using a transparent glass slide to compress and reduce the lesion thickness facilitating the laser action [19, 20]. A scanning movement is used to deliver the laser energy without keeping the fiber fixed for more than 5-10 seconds in order to avoid excessive thermal effects. Due to the high absorption of diode laser energy by the blood into the lesion, it becomes smaller and lighter during the treatment; this effect is called “forced dehydration” [21].

Apfelberg introduced the treatment of deep vascular lesions using ILP, and due to the noted successful outcomes, many surgeons started using this technique [22].

In the ILP technique, the laser fiber is used to deliver the laser energy directly into the depth of the lesion, in order to coagulate the deep lesion components and preserve the mucosal or cutaneous surface.

Despite the deep tissue absorption of the diode laser, the deep layer cannot be reached using both techniques TMT and LT in deep and large lesions, since the laser tissue penetration is limited [23–25].

In this clinical case, laser treatment using TMT induced excessive heat absorption due to the irradiation of the entire lesion in the same session, causing postoperative side effects such as severe pain, swelling, and ulcer formation.

Large lesions should be treated in more than one session, achieving a reduction of the lesion size and evaluating which laser technique should be used for each session to obtain the complete resolution of the lesion [26].

## 5. Conclusion

In the oral cavity, ulcer formation after laser treatment should not be neglected since it can lead to temporary trismus and may reduce both the mobility of the tongue and the depth of the oral vestibule.

It was recommended to use LT in order to avoid any invasive and severe side effects and to obtain a satisfactory result.
